# Pinpointing dynamic coupling in enzymes for efficient drug design

**DOI:** 10.4155/fsoa.2015.0017

**Published:** 2016-01-25

**Authors:** E Joel Loveridge, Rudolf K Allemann

**Affiliations:** 1 School of Chemistry, Cardiff University, Park Place, Cardiff CF10 3AT, UK

**Keywords:** chemical ligation, enzyme catalysis, isotope substitution, protein dynamics

**Figure 1.**
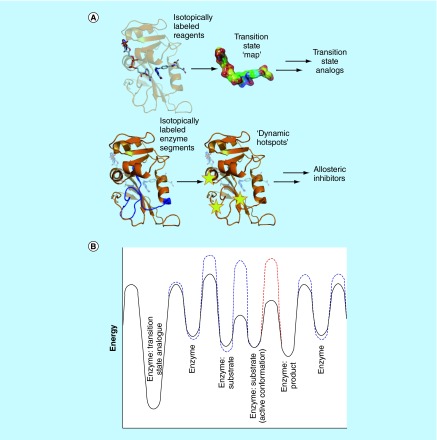
**Complementary approaches to enzyme inhibition.** **(A)** Use of isotope labeling methodologies to generate transition state analogues and allosteric inhibitors. **(B)** Energy landscape of a model enzymatic reaction (black) showing changes in the presence of allosteric inhibitors targeting conformational changes (blue) and transition state formation (red).

Enzymes are proteins that catalyze almost every chemical reaction in living systems, achieving rate enhancements of up to 21 orders of magnitude relative to the uncatalyzed reactions. However, despite a century of intense investigation, the biophysical basis of the enormous catalytic power of enzymes is not completely understood. Enzymes are not only central to living systems, but also to many industrial processes such as the production of food, textiles, detergents, pharmaceuticals and other chemicals where environmentally friendly, green methods are of ever increasing importance. Because of their central role for life, enzymes are key drug targets and enzyme inhibition is a central strategy in the design of new drugs. Acetylsalicylic acid, azidothymidine, acyclovir, allopurinol, chloramphenicol, exemestane, fosfomycin, isoniazid, methotrexate, profens, proguanil, statins, thiouracil and warfarin are but a small subset of approved drug substances that are used in the clinic to treat, among others, pain, fever, inflammation, malaria, cancer, HIV, bacterial and viral infections, rheumatoid arthritis, osteoarthritis and heart disease, through the inhibition of key enzymes.

In 1894 Emil Fischer (Nobel Prize in Chemistry 1902) proposed his lock-and-key model, which simply states that an enzyme's active site complements its substrate in the same way as a lock complements its key, as a rationale for the catalytic power of enzymes [1]. This was adapted in 1930 by John Burdon Sanderson Haldane to include ground state destabilization (changes to the structure of the ‘key’ when it enters the ‘lock’) [2]. Linus Pauling (Nobel Prize in Chemistry 1952) incorporated transition state theory (TST), which had earlier been developed by Michael Polanyi and extended by Henry Eyring [3–5]. In TST, enzymatic rate accelerations are explained by the lowering of the transition state energy and/or increase of the ground state energy. TST has underpinned the design of transition state analogues for enzyme inhibition and the creation of catalytic antibodies. The rationale behind transition state analogues is that if enzymes have evolved to bind tightly to the transition state of a reaction then a stable but unreactive molecule that resembles this transition state will be a good inhibitor of the enzyme. Although the transition state is only transiently formed during the reaction and is not directly observable, information on its structure and electronic state may be probed by kinetic isotope effects, the ratio of the rate constants was obtained when isotopically labeled reactant molecules are used and those seen using reactant molecules with isotopes of natural abundance [6,7].

These theories did not incorporate dynamics, despite the fact that enzymes are highly flexible molecules. Daniel E Koshland's induced fit hypothesis accounted for large-scale changes in enzyme structure on binding a substrate [8], and many enzymes adopt different conformations as they move through their catalytic cycles [9]. Because the electrostatics of an enzyme determine the free energy surface, equilibrium motions reflect the change in interactions between the atoms as the enzyme moves across the free energy surface. Therefore, while it is generally accepted that electrostatic preorganization plays the dominant role in enzyme catalysis [10], studies of the motions of an enzyme provide central information about the nature of its energy ‘landscape’.

Recently, Vern Schramm's laboratory at Albert Einstein College of Medicine, New York, pioneered the use of ‘heavy enzymes’ to probe enzyme dynamics. In their approach >98% of the carbon, nitrogen and nonexchangeable hydrogen atoms of an enzyme are replaced with the corresponding stable heavy isotopes ^13^C, ^15^N and ^2^H, leading to a molecular weight increase of ˜10%, and the catalytic properties of this heavy enzyme are compared with the natural light version [11,12]. Isotope substitution does not change the chemistry of the catalyzed reaction but slows motions of the catalyst, from femtosecond bond vibrational frequencies through millisecond conformational changes to slow domain rearrangements. Hence, the entire profile of motions of an enzyme can be altered and investigated by isotopic substitution. The ratio of the reaction rate constants of the ‘heavy’ enzyme and the ‘light’ enzyme therefore provides information on the degree of motional coupling to the reaction.

We have applied heavy enzyme methodology to the reaction catalyzed by the enzyme dihydrofolate reductase (DHFR), a vital biosynthetic enzyme required for the production of DNA and a number of amino acids. By combining experimental heavy enzyme measurements to detect motional coupling with computational studies to understand the underlying molecular mechanism, we have demonstrated not only that slower DHFR motions couple to substrate binding and product release, which showed that these motions are important for progression through the catalytic cycle [13,14], but also that femtosecond bond vibrations couple directly to transition state formation [15–18]. However, we found the coupling of bond vibrations to the actual chemical reaction to be detrimental for DHFR catalysis [15]. Indeed, dihydrofolate reductases from across a range of organisms appear to minimize fast dynamic coupling under conditions most representative of their physiological niches, but with elevated dynamic coupling seen under nonphysiological conditions [18] and in variant enzymes [16]. These apparently contradictory results demonstrate the delicate nature of motional coupling to catalysis. We have refined this global picture of motional coupling by isotopically labeling specific segments of an enzyme and therefore experimentally determining which parts of the enzyme show motional coupling [19]. Two isotopic hybrids of DHFR were prepared by chemical ligation techniques [20], one in which the mobile N-terminal segment contained heavy isotopes while the remainder of the protein was of natural isotopic abundance, and one in which only the C-terminal region was isotopically labeled. These experiments revealed that the N-terminal region is only involved in beneficial millisecond conformational motions, while in the C-terminal region fast dynamic motions couple unfavorably to barrier crossing, thereby slowing the reaction [19]. The combination of chemical ligation and heavy enzyme approaches has shown for the first time that different parts of an enzyme affect different aspects of its function. Schramm has recently used labeling methodology to determine which amino acid residue types have motions that couple to the reaction [21].

Much has been learned about Nature's catalysts but much remains to be discovered before we can claim to fully understand the physical basis of enzyme catalysis. We are however now in a position where results from fundamental investigations can be used to drive the development of novel chemicals of societal use. Using kinetic isotope effects based on labeling the reactant molecules, Schramm *et al*. have mapped the transition state of purine nucleoside phosphorylase, a key enzyme involved in DNA recycling [22]. Synthesis of molecules based on this work has led to the production of stable transition state analogues with several million-fold tighter binding to the enzyme than its natural substrates, and excellent selectivity between the enzymes from human and the malaria parasite *Plasmodium falciparum* [23,24]. Heavy enzyme methodology, based on labeling of the enzyme itself, has revealed the involvement of fast enzyme motions in forming the transition state of the reaction [11]. These two complementary techniques each provide a potential route to new enzyme inhibitors that may be used as drugs (Figure 1).

The central role of DHFR in metabolism means that it has long been a valuable target for drugs such as the antibacterial trimethoprim, the antimalarial pyrimethamine and the clinically highly successful anticancer agent methotrexate. DHFR has recently attracted renewed attention to overcome resistance now seen against existing drugs and to reduce side-effects by increasing selectivity. It has been suggested that inhibitors of enzyme motions crucial for an enzyme's catalytic cycle can be designed and used as new therapeutics [25]. Therefore, in addition to designing transition state analogues based on traditional kinetic isotope effect methods, the combination of chemical ligation and heavy enzyme approaches may allow the construction of a new tool for drug design and development, as understanding the nature and location of coupling between the motions of an enzyme and bound molecules will assist in the design of ‘allosteric inhibitors’ that bind to more remote regions of the enzyme but prevent motions important for the catalytic cycle (Figure 1). Whereas a transition state analogue places the enzyme in an energy well too deep for it to readily escape, an allosteric inhibitor alters the energy barriers to conformational change and so blocks productive progression through the catalytic cycle. Because heavy enzyme approaches can distinguish motions that are involved solely in conformational changes from those involved in transition state formation [19], the two classes of motion may be targeted separately (Figure 1). More generally, the combination of chemical ligation and heavy enzyme approaches can be applied to a wide range of pharmaceutically and industrially important enzymes, and will lead to new candidates for biological and medical applications, as well as new production routes for enzymes of industrial use. Most importantly, it demonstrates how bringing together different experimental and computational methods to address some of the most challenging problems in modern enzymology can generate applications in medicine with wide societal benefits.
